# Improvement of I'mRT MatriXX in terms of spatial resolution and large area acquisition for patient-specific intensity-modulated radiotherapy verification

**DOI:** 10.4103/0971-6203.54850

**Published:** 2009

**Authors:** Arun S. Oinam, Lakhwant Singh, S. C. Sharma, Pradeep Goswami

**Affiliations:** Department of Radiotherapy, PGIMER, Chandigarh, India; 1 Department of Physics, Guru Nanak Dev University, Amritsar, India

**Keywords:** 2D array ion chambers, convolution, intensity-modulated radiotherapy, multiple-sequence acquisition

## Abstract

2D array of ionization chambers can be used for both absolute and relative dose verification of patient-specific intensity-modulated radiotherapy (IMRT) quality assurance. After an analysis of the dose linearity and spatial resolution of this 2D array (I'mRT MatriXX), the signal sampling time of 200 ms was selected for data acquisition. Multiple-sequence acquisitions at the nearest 4 positions with the shift of half of the distance between the centers of two adjacent ion chambers increase the spatial resolution up to four times when used with this I'mRT MatriXX. IMRT verification of head-and-neck case, which requires a large area for dosimetric verification, can be done with limited size of 24×24 cm^2^, depending on the user requirements. It is found that the convolution method can also be used to improve the IMRT dose verification with the same parameters of the passing criteria significantly, viz., up to 99.87% agreement, by smoothening the treatment planning system profile.

## Introduction

Two-dimensional (2D) arrays of ionization chambers or diodes have been used recently for the routine relative and absolute planar dose verification of IMRT plans to simplify and reduce the quality assurance (QA) workloads of physicists in a busy department.[1–3] The spatial resolution of 2D arrays of ion chambers or diodes was limited by the size and the distance between the centers of adjacent ion chambers. Different techniques[3–5] have been evolved to improve the spatial resolution of 2D arrays of ion chambers. Spezi *et al.*[3] reported that the spatial resolution of these 2D arrays systems can further be increased by using multiple-acquisition sequence of IMRT verification by positioning 2D arrays of ion chambers at different positions. The authors of this paper have found that the image resolution of this standard acquisition mode can be increased up to four times. Björn Poppe *et al.*[4,5] have also developed an optimization criterion of sampling step width frequency of 0.2 mm^−1^ for this spatial resolution. However, these papers do not mention about the optimization of the spatial resolution of 2D arrays of ion chambers matrix (I'mRT MatriXX) procured from Scandetronix Wellhofer, Germany. In this present paper, we have attempted to improve in terms of spatial resolution and region of interest of large field data acquisition of 2D arrays of ion chambers matrix used in routine patient-specific quality assurance test. Moreover, the optimization criterion of this I'mRT MatriXX has also been reported in the present work.

## Materials and Methods

### 2D array of ion chamber matrix and dose measurement

**Figure 1:**
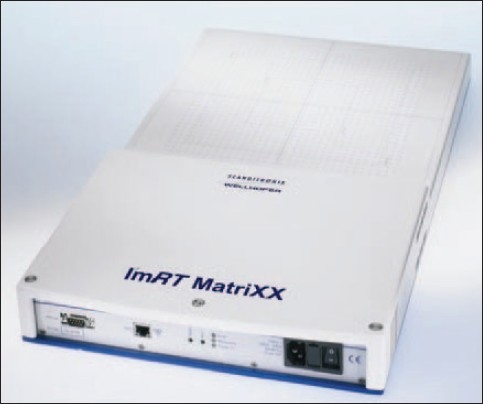
2D array ion chamber matrix (I'mRT MatriXX of Scanditronix Wellhofer, Germany)

2D array of ion chamber I'mRT MatriXX [Figure 1], procured from Scanditronix Wellhofer, Freiburg, Germany, consists of 1020 ion chambers, which are arranged in 32×32 matrix. Each detector has a diameter of 4.5 mm, height of 5 mm and sensitive volume of 0.08 cc. The distance between two adjacent detectors measured from center to center is 7.62 mm. The thickness of the ridge between two adjacent detectors is 3.12 mm. The sensitive area for dose measurement of 2D ion chambers array is 24×24 cm^2^ and is operated at a potential of 500 V. All the measurements were performed using 6 MV photon beams of a medical linear accelerator (Clinac DHX, Varian Medical Systems, Palo Alto, CA, USA). This linear accelerator is equipped with a dynamic multileaves collimator (MLC) consisting of 40 pairs of leaves and having projected leaf width of 1 cm at isocenter. The intensity-modulated radiotherapy (IMRT) fields of 6 MV photon beams, computed with ECLIPSE^®^ treatment planning system (TPS), version 8.05, Varian Medical Systems, Palo Alto, CA, USA, were used for IMRT verification. The intensity fluences of these IMRT fields were optimized using HELIOSE^®^ optimization software (DVO version 8.05).

### Study of lateral response of a single ionization chamber of I'mRT MatriXX

Radiation beam of 2 mm slit opening at isocenter of dose-dynamic MLC was used to determine the lateral response of a single ionization chamber of 2D arrays of ion chambers matrix. To reduce the collimator scattering factor to as low as possible, Y jaws were opened 5 mm asymmetrically and X jaws were opened 20 cm symmetrically over an array of ion chambers. Beam-sweeping fields of 2 mm slit opening using dynamic MLC dose delivery files with MLC movement along the edge of Y jaws from bank A to B were exposed on this 2D array of chambers. The radiation exposed on the ion chambers array was 500 monitor units at the dose rate of 300 monitor units per minute. The radiation data was acquired at 10 cm depth in movie mode. These single-snap data of an ion chamber in movie mode can be utilized for finding the lateral response of a single ionization chamber. The time of acquisition for each snap was varied from 100 to 300 ms in steps 50 ms. The signals acquired using different acquisition times for each snap can be studied in terms of Fourier transformation frequency for obtaining of optimum acquisition times. The single-snap profiles were transferred to MATLAB platform in ASCII format. The response of a single ionization chamber with respect to the position of MLC slit of 2 mm opening was analyzed using Fourier transformation.

### Dose linearity with different sampling times

Open beam of 20×20 cm^2^ was acquired with different sampling times, from 100 to 300 ms in steps of 50 ms. Dosimetric calibration of I'mRT MatriXX was done in single-snap mode with 100 cGy delivered at 10 cm depth in solid water phantom. Doses delivered were within the range of 10 to 600 cGy. The step for dose delivered for linearity checking was 50 cGy, from 50 to 400 cGy.

### Improvement of I'mRT MatriXX using multiple acquisitions

#### Spatial resolution

Multiple acquisitions of dose profiles at 10 cm depth for an IMRT verification field were taken using two-dimensional arrays of ion chamber matrix (I'mRT MatriXX). Four different positions of 3.8 mm shift from isocenter perpendicular and parallel to MLC leaves were chosen for these acquisitions. The distance between the midpoint of the two adjacent ion chambers was 7.61 mm, and that between edge and midpoint of ion chamber was 3.8 mm. These integral 2D dose profiles of I'mRT MatriXX acquired at these different positions were exported in ASCII format. A program was written in MATLAB^®^ to process these dose profiles to produce a resultant 2D dose profile. This program shuffle matrix elements of four matrices which were acquired at the above four different positions resulted in an increase of spatial resolution up to four times. If a_ij_, b_ij_, c_ij_ and d_ij_ are the i^th^ row and j^th^ column of four matrices A, B, C and D resulted from the multiple-sequence data acquisitions by 2D array ion chamber matrix at four different positions, then the resultant matrix R after doing matrix suffles is calculated as

(1) $$R_{\mathrm{ij}}=\left[\begin{array}{cc} a_{\mathrm{ij}} & b_{\mathrm{ij}}\\ c_{\mathrm{ij}} & d_{\mathrm{ij}} \end{array}\right]$$

where i and j go from 1 to 32 and R_ij_ is the i^th^ row and j^th^ column matrix element which consists of another submatrix of elements a_ij_, b_ij_, c_ij_ and d_ij_…

(2) $$R_{\mathrm{mn}}=R_{\mathrm{ij}}$$

and m and n go from 1 to 64.

This MATLAB program suffles the matrix elements according to Appendix 1.

Multiple acquisition of dose profile at 10 cm depth was done to cover the entire region of interest by positioning 2D arrays of ion chambers matrix at different positions. The position of I'mRT MatriXX for data acquisition was noted down for processing of 2D dose profiles, and these two matrices were merged to produce the resultant 2D dose profile. If A and B are two matrices with i^th^ row and j^th^ column elements a_ij_ and b_ij_, respectively, then the resultant matrix R is found out as

(3) R=[AB]

(4) $$R_{\mathrm{mn}}=[[a_{\mathrm{ij}}][b_{\mathrm{ij}}]]$$

where i and j go from 1 to 32 and m goes from 1 to 64 and n from 1 to 32. The merging of the two matrices is shown in Appendix 2.

#### Convolution kernel for the comparison of 2D profiles

Point spread kernel function (probability density function) was reconstructed with a Gaussian function from lateral response function of snap data acquisition with sample time ranging from 100 to 300 ms in steps of 50 ms.

(5) $$G(x,y,)=\frac{1}{\sigma \sqrt{2\sigma }}.e^{\left(\frac{x^{2}+y^{2}}{2\pi^{2}}\right)}$$

where σ is standard deviation of lateral response profile, x and y are the coordinates within the search distances of 10 mm in the matrix size of 10×10.

The TPS profiles were convolved with the point spread function G (x,y). It is expressed mathematically as

(6) $$\mathrm{T\prime}(x,y)=\begin{array}{c} \infty \\ \int \\ \infty \end{array}\begin{array}{c} \infty \\ \int \\ \infty \end{array}T(u,v)G(u-x,v-y)\mathrm{dudv}$$

where T(x,y) and T'(x,y) are 2D TPS profiles and convolved 2D TPS profiles, respectively. The convolved TPS profile is written symbolically as

(7) T'(x,y)=T(x,y)⊗G(x,y)

Whereas in terms of 2D convolution of two discrete input matrices of matrix T having dimensions (Ma, Na) and matrix G having dimensions (Mb, Nb), the equation of the output matrix of 2D discrete convolution is given by

(8) Tapos(i,j)=Σm=0(Ma-1)Σn-0(Na-1)T(m,n)*G(i-m,j-n)

where 0 ≤i <*Ma*+*Mb*-1 and 0 ≤j<*Na*+*Nb*-1

**Figure 2:**
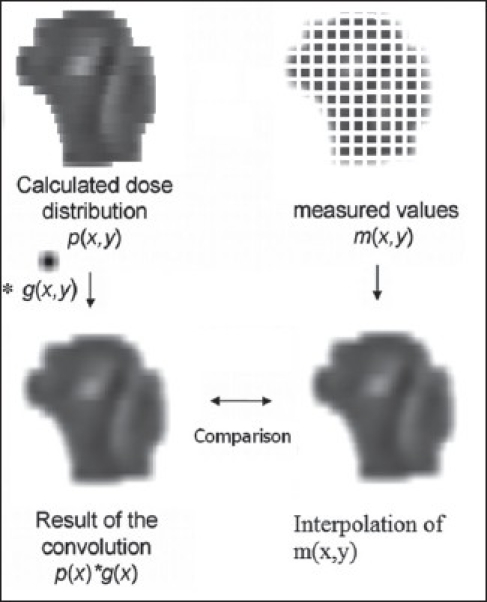
The steps of dosimetry verification technique using convolution

The measured ion chamber profile, M (x,y), was interpolated linearly with grid size of 1×1 mm^2^. Then these processed profiles [T'(x,y) from equations 7 and 8] and measured profiles [M (x,y)] were compared in OmniPro-IMRT software of Scandetronix Wellhofer, Germany, according to the steps shown in Figure 2. The gamma values were then calculated to compare these two profiles, as reported by Low *et al.,*[6] with 3% dose difference and 3 mm distance-to-dose agreement.

## Results and Discussion

**Figure 3:**
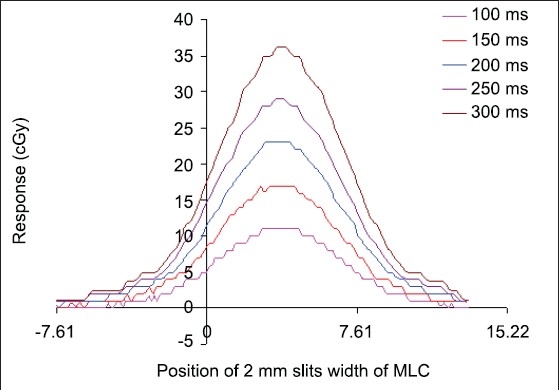
Lateral response profiles (dose profiles) of single ion chamber for different sampling times at different positions of 2-mm slit width of multileaves collimator (MLC).

**Figure 4:**
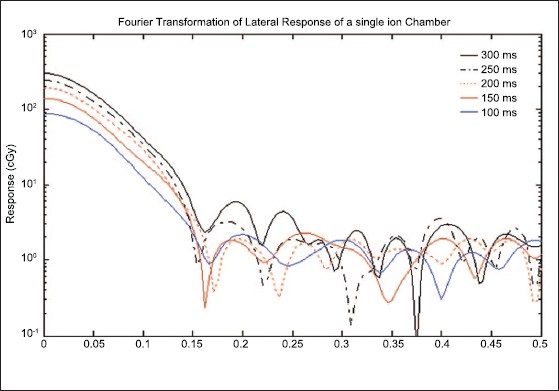
1D Fourier transformation of lateral response function for different sampling times of Figure 1. Continuous deep blue, brown and black color curves represent the 1D Fourier signal profiles of sampling time 100, 150 and 300 ms, respectively. Similarly discontinuous orange and black color curves represent those of 200 and 250 ms, respectively

The lateral response of a single ion chamber for different sampling times is shown in Figure 3. A prominent peak appearing in this figure can be utilized for the improvement of resolution of 2D arrays ion chamber matrix. The radiation dose delivered on the 2D ion chamber arrays with beam-sweeping fields of 2 mm MLC slit opening produces the changes in the relative response of a single ion chamber due to the different positions of MLC slit. The variation of relative response was characterized by standard deviations of 0.356, 0.354, 0.354, 0.353 and 0.347, respectively, for the different signal sampling times of 100, 150, 200, 250 and 300 ms. The maximum SD of 0.356 was found for sampling time of 100 ms while with minimum standard deviation of 0.347 for the sampling time of 300 ms. The variations of relative response were approximately equal for all the sampling times but were larger for smaller sampling times. The spatial spread, that is, full width at half maximum value (FWHM) of signal response, of each sample is also the same for all the signal samples times except a small difference at the peak, which is flattened in case of small sampling time and sharp for larger sampling time, as shown in Figure 3. We have used Fourier transformation technique to determine the differences between these peaks. The Fourier transformations of lateral response profiles are shown in terms of spatial frequency (mm^−1^) and amplitude (cGy) of Fourier signal [Figure 4]. The first zeros of each Fourier signal for different signal sampling times of 100, 150, 200, 250 and 300 ms were found at spatial frequency of 0.168, 0.163, 0.171, 0.154 and 0.163 mm^−1^, respectively [Table 1]. These differences could not be visualized with a simple Cartesian coordinate and dose response peaks of lateral response profiles. The first zero spatial frequency of 200 ms sampling time produced the largest spatial frequency (0.171 mm^−1^). The largest spatial frequency of first zero corresponds to better spatial resolution according to Fourier transformation. This shows that the sampling time of 200 ms provides better spatial resolution, though this 2D array ion chamber works with zero dead time of signal sampling. Poppe *et al.*[4,5] reported that the lateral response of PTW 2D-Array, 10024 version of PTW Freiburg, Germany, was a trapezium of 5 mm top and 9 mm base. We found that the first zero of this lateral function was found at almost the same spatial frequency of 0.144 mm^−1^ as reported by Poppe *et al.*[4,5]

**Table 1:** The first zeros of Fourier signals and the variation of relative responses of lateral response of a single ion chamber for different sampling times

*Sampling Time*	*Spatial frequency (mm^−1^)*	*Standard Deviation*
100 ms	0.168	0.356
150 ms	0.163	0.354
200 ms	0.171	0.354
250 ms	0.154	0.353
300 ms	0.163	0.347

The linearity of 2D array ion chambers from absorbed dose of 10 to 600 cGy was measured for different signal sampling times, starting from 50 to 400 ms. The nonlinearity values were higher for lesser sampling time and decreased with increase of sampling time. These values were 0.66%, 0.55%, 0.3925%, 0.237% and 0.1277% for signal sampling times of 50, 100, 200, 300 and 400 ms, respectively [Table 2]. This shows the improvement in linearity with increase in sampling time. Although there is an improvement in linearity with increase in sampling time, yet spatial resolution becomes poor at higher as well as lower sampling times. The optimum sampling time for signal acquisitions was found to be 200 ms.

**Table 2:** Dose linearity of different sampling times

*Sampling Time*	*Non Linearity %*
50 ms	0.66
100 ms	0.55
200 ms	0.39
300 ms	0.237
400 ms	0.13

**Figure 5:**
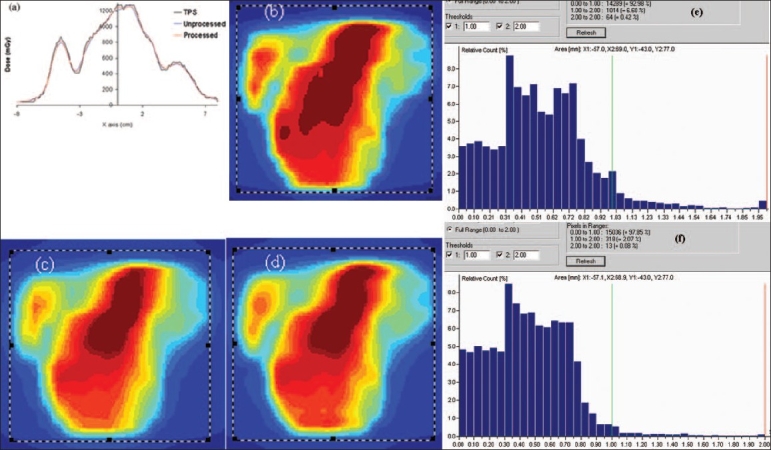
2D profile comparison of TPS, unprocessed profile of I'mRT MatriXX without any shift of position and processed profile of I'mRT MatriXX using multiple profiles acquisition at four different positions [(0,0); (3.81 mm, 0); (0, 3.81 mm) and (3.81 mm, 3.81 mm)]. a represents the 1D profile comparison of TPS 2D dose map (b), unprocessed (c) and processed (d) 2D dose maps of I'mRT MatriXX. Improvement of gamma histogram evaluation (delta distance = 3 mm and delta dose = 3%) by 5% from (e) TPS vs. unprocessed 2D profile to (f) TPS vs. processed 2D profile

**Figure 6:**
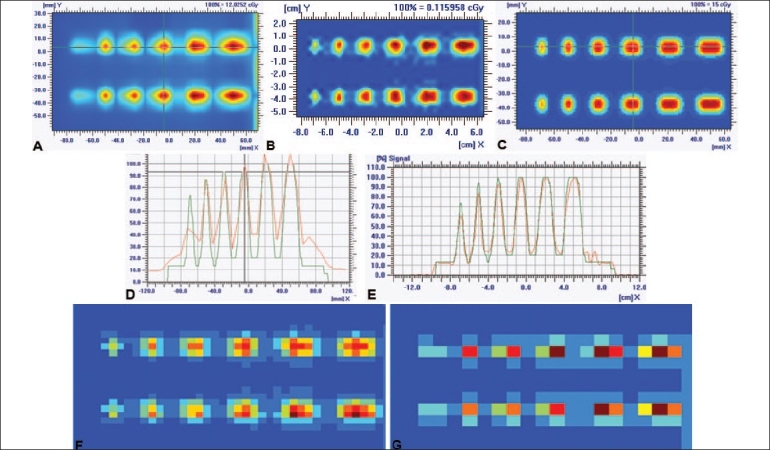
Comparison of 2D plane dose profiles of peak test between unprocessed profile of I'mRT MatriXX (A), processed profile of I'mRT MatriXX (B) and TPS (C). D represents the 1D profile comparison between TPS and unprocessed matrix data. E represents the 1D profile comparison between TPS and processed matrix data of I'mRT MatriXX with 4 adjacent shifts, showing the improvement of spatial resolution by detecting the 2.5 mm MLC gap, which cannot be resolved by unprocessed matrix data (D). Uninterpolated matrix data of processed profile (F) shows the increase of spatial resolution to up to 4 times as compared with that of unprocessed profile (G).

Figure 5A shows the comparison of a single-field IMRT verification using multiple-sequence acquisition technique with the conventional method of IMRT verification. The multiple IMRT data was acquired at four different positions by shifting the 2D array by half the distance between the two adjacent chambers. The matrix elements of these four matrices were merged and suffled to obtain the processed profiles. Figure 5A shows the comparison of 1D profiles amongst the 2D plane dose profiles of TPS [Figure 5B], processed profile [Figure 5C] using multiple-acquisition technique and unprocessed profile [Figure 5D] with the conventional IMRT verification method. This showed the improvement of processed profile, which is closer to TPS profile. The same improvement was also observed with gamma evaluation histograms [Figures 5D, 5E]. The gamma evaluation of TPS profile versus processed 2D profile using 3% dose tolerance (ΔD) and 3 mm distance-to-dose agreement (Δd) within a region of interest reveals that the pixel population within the signal range from 0 to 1.0 was 97.85% as compared with 92.98% for TPS profile versus unprocessed 2D plane dose profile within the same region of interest. Figure 6 shows the comparison between 2D plane profiles of TPS versus processed profile and TPS profile versus unprocessed profile for benchmark IMRT field (peak test; dynamic MLC dose delivery file which is generated with different MLC gaps, ranging from 2.5 to 15 mm in steps of 2.5 mm). This figure shows improvement in resolution up to 4 times of conventional IMRT verification with I'mRT MatriXX. The improvement in dosimetry verification can be clearly observed in Figures 6D and 6E, which show that processed 1D profile is closer to TPS profile [Figure 6D] as compared to unprocessed 1D profile [Figure 6E]. The gamma evaluation of TPS profile against processed 2D profile using ΔD of 5% and Δd of 3 mm within a region of interest calculates the pixel population within the signal range from 0 to 1.0 as 94.32% as compared with 58.29% for TPS profile versus unprocessed 2D plane dose profile. So the multiple-sequence acquisition technique improves the spatial resolution of IMRT dose verification, as reported by Emialano *et al.*[3]

**Figure 7:**
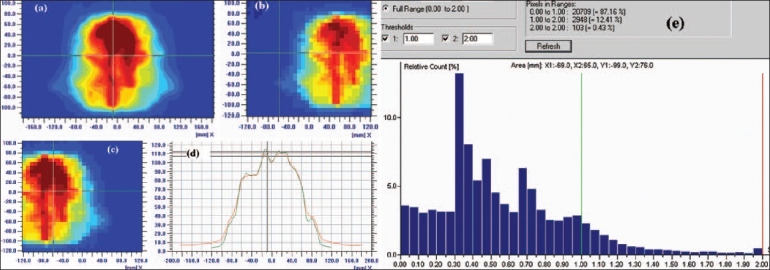
IMRT verification of a head and neck patient, which requires a large region of interest for dose verification. 2D dose profile (a) of large region, viz., 24×37 cm^2^, was reconstructed from two 2D dose profiles (b) and (c) of area 24×24 cm^2^ acquired by positioning the isocenter at ± 6.5 cm from matrix center. (d) 1D profile comparison between TPS and processed large region 2D array matrix data. (e) the gamma histogram for comparison of 2D dose profiles between TPS data and processed large region 2D array data using gamma evaluation (delta distance = 3 mm and delta dose = 3%) method

Figure 7 shows the results of the technique for large area IMRT dose verification of radiotherapy treatment. 2D profile within the region of interest 24×37 cm2 cannot be acquired with the present I'mRT MatriXX maximum area (24×24 cm^2^) of 2D dose profile. This 2D dose profile of area 24×37 cm^2^ was reconstructed from two 2D dose profiles [Figures 7B and 7C] of area 24×24 cm^2^ acquired by positioning the isocenter at ± 6.5 cm from matrix center. Figure 7D represents 1D profiles comparison between TPS and processed large region 2D array matrix data, and this shows the increase of profile axis from 24 to 37 cm. Figure 7E shows the gamma histogram of the 2D dose profiles comparison between TPS data and processed large region 2D array data using gamma evaluation method of passing criteria of ΔD of 3% and Δd of 3 mm. 1D and 2D profiles comparison shows the increase of the range of x-coordinate of Matrix profile.

**Figure 8:**
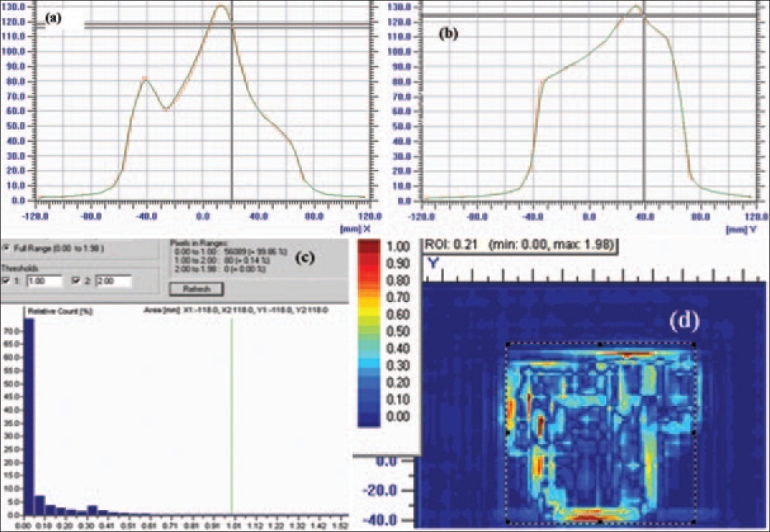
2D dose profile comparison between convolved TPS profile and I'mRT MatriXX profile. (a) 1D profile comparison between I'mRT MatriXX and convolved TPS along x-axis and (b) 1D profile comparison between I'mRT MatriXX and convolved TPS along y-axis. Gamma histogram (c) of gamma values (d) depicted with color spectrum within the region of interest of 2D plane using delta dose 3% and delta distance 3 mm

Figure 8 shows the results of IMRT verification using convolution technique. Here the TPS 2D plane dose profile T(x,y) was convolved with a point spread function, also called Gaussian function, G(x,y), rather than using the rectangular lateral response function of Poppe *et al.*[4,5] This scattering kernel is generated to conform to the shape of lateral response profile of a single ion chamber. Then, this convolved profile was compared with I'mRT MatriXX–measured data after interpolating to a higher resolution of 1×1 mm^2^ grid size. Figure 8A shows 1D profile comparison between convolved TPS 2D profile and interpolated 2D profile of I'mRT MatriXX along x-axis. Similarly Figure 8B shows 1D profiles comparison along y-axis. It is clearly observed that convolved TPS profile is very close to I'mRT MatriXX-measured data. Figure 8C shows the gamma histogram calculated using ΔD of 3% and Δd of 3 mm. In the region of interest as shown in Figure 8D, the population of pixel of the gamma values, which is depicted by color spectrum within the signal range from 0 to 1, was 99.86%. This shows drastic improvement in dosimetric verification of IMRT for routine patient-specific quality assurance using convolution technique.

## Conclusion

The larger the acquisition times, the poorer the resolutions. If the acquisition time was reduced, the resolution was found to improve. But the dosimetric nonlinearity hampered the resolution. From this study, we conclude that the signal sampling time of 200 ms is the optimum criterion for data acquisition of dynamic IMRT verification using I'mRT MatriXX. Using this parameter, we performed different IMRT verification techniques. Multiple-sequence acquisitions at the nearest four positions with a shift of half of the distance between the centers of two adjacent ion chambers increase the spatial resolution to up to four times with I'mRT MatriXX. IMRT verification of large field can be done with limited size, viz., 24×24 cm^2^, depending on the user requirements. The convolution method can also improve the IMRT dose verification significantly without changing the values of passing criteria of IMRT verification. All these techniques can be applicable in a simple program, developed in a MATLAB software.

## Appendix 1

If X(x,y), Y(x,y), Z(x,y) and Q(x,y) are the measured four matrices (m×n matrix elements) using 2D array ionchamber at four different shift positions, then the resultant profile of matrix data of size 2m×2n were reconstructed by merging and suffling these four matrices as:

Resultant (x,y)=[X11 Y11 X12 Y12……Z11  Q11 Z12 Q12……X21  Y21 X22 Y22……Z21  Q21 Z22 Q22………………………………]2m×2n,

where X(x,y)=[X11 X12……Y(x,y)=[Y11 Y12……X21 X22……Y21 Y22……………………]m×n………………]m×n

Z(x,y)=[Z11 Z12……Q(x,y)=[Q11 Q12……Z21 Z22……Q21 Q22……………………]m×n………………]m×n

## Appendix 2

If X(x,y) and Y(x,y) are the measured two matrices (m×n matrix elements) using 2D array ion chamber at two different positions, then resultant matrix profile of size m×(n+1) were reconstructed by merging these two matrices as:

Resultant (x,y)=[X11 X12 X13 Y11 Y12 Y13……X21  X22 X23 Y21 Y22 Y23……X31  X32 X33 Y31 Y32 Y33…………………………………………]m×(n+1),

where X(x,y)=[X11 X12 X13……X21  X22 X23……X31  X32 X33…………………………]m×n

where Y(x,y)=[Y11 X12 Y13……Y21  Y22 Y23……Y31  Y32 Y33…………………………]m×l
